# Supplementary material for: Complex‐centric proteome profiling by SEC‐SWATH‐MS
Source: Mol Syst Biol. 2019 Jan 14;15(1):e8438. doi: 10.15252/msb.20188438 (PMC6346213; doi:10.15252/msb.20188438)
Supplement: Supplementary file 8 — Dataset EV7 [file MSB-15-e8438-s008.zip › feature_plots_string/Q86Y82.pdf]

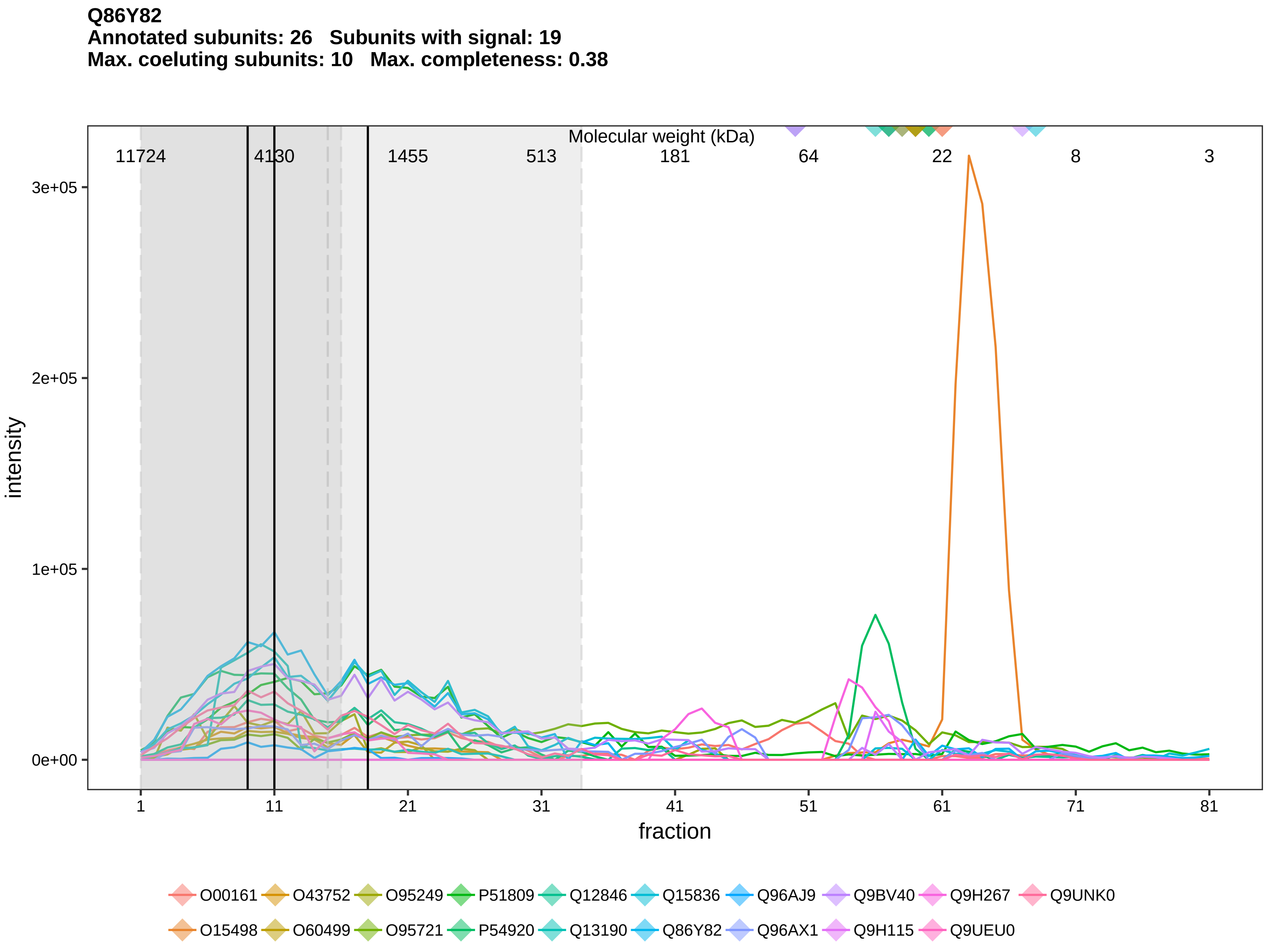

Q86Y82
Annotated subunits: 26 Subunits with signal: 19
Max. coeluting subunits: 10 Max. completeness: 0.38
Molecular weight (kDa)
11724
4130
1455
513
181
64
22
8
3
3e+05
2e+05
1e+05
0e+00
intensity
1
11
21
31
41
51
61
71
81
fraction
O00161
O43752
O95249
P51809
Q12846
Q15836
Q96AJ9
Q9BV40
Q9H267
Q9UNK0
O15498
O60499
O95721
P54920
Q13190
Q86Y82
Q96AX1
Q9H115
Q9UEU0
